# The College Studentships

**Published:** 1920-10-16

**Authors:** 


### The College Studentships.

The result of the studentship examination which we give in another column (p. vi.) has been awaited with much interest. The final awards are made somewhat after the example of the Appointments Bureau at the universities, precluding all possibility of the mere brain-worker and memoriser being preferred before the woman with the higher qualities needed in the good nursing sister. Brains are essential, for there is no woman quite so incapable as the stupid nurse. But a large heart is even more to be desired, with all the faculties which go with that happy possession.

In order that they may judge of both heart and head, the committee have before them not only the papers in the written examination but also the nurse's record of work performed and qualities displayed in her training-school. It may be surmised that there is seldom any great discrepancy between the evidence of the examination tests and that of the previous career, for the questions are of a nature to elicit answers which should throw considerable light on the character. However this may be, it remains a cardinal principle of the selection that the student shall have given evident tokens of possessing a true vocation before she advances to the stage in her career in which she will exercise widespread influence on her fellow nurses.

The good which these scholarships are doing is not to be measured by their effect on the successful students. They are rousing a new spirit in the training-schools. The old unruffled surface of training, confined to routine subjects and almost specialising in narrowness, has already broken into a thousand cross-currents of interest in outside affairs. Wherever a nurse is preparing to compete there is expectation, wider preparation, a sense of the great public for which nurses exist and out of which they are chosen. In short the scholarships make for that quickened sense of social values the absence of which in the training-schools has so long been deplored.

